# Neurogenesis of Retinal Ganglion Cells Is Not Essential to Visual Functional Recovery after Optic Nerve Injury in Adult Zebrafish

**DOI:** 10.1371/journal.pone.0057280

**Published:** 2013-02-20

**Authors:** Suqi Zou, Chen Tian, Shuchao Ge, Bing Hu

**Affiliations:** CAS Key Laboratory of Brain Function and Disease, School of Life Sciences, University of Science and Technology of China, Hefei, Anhui, P. R. China; Schepens Eye Research Institute, Harvard Medical School, United States of America

## Abstract

Zebrafish central nervous system (CNS) possesses a strong neural regeneration ability to restore visual function completely after optic nerve injury (ONI). However, whether neurogenesis of retinal ganglion cell (RGC) contributes to functional recovery remains controversial. Our quantitative analysis of RGCs in different ONI models showed that almost all RGCs survived in optic nerve crush (ONC) model; while over 90% of RGCs survived in the first 2 weeks with 75% remaining after 7 weeks in optic nerve transection (ONT) model. Retrograde labeling from tectum revealed a surprising regeneration rate, with over 90% and over 50% of RGCs regrowing axons to tectum at the first week in ONC and ONT model respectively. In the latter one, the number of regenerative RGCs after 4 weeks had no significant difference from the control group. As for neurogenesis, newborn RGCs were rarely detected either by double retrograde labeling or BrdU marker. Since few RGCs died, microglia number showed a temporary increase at 3 days post injury (dpi) and a decrease at 14 dpi. Finally, myelin structure within retina kept integrity and optomotor response (OMR) test demonstrated visual functional restoration at 5 weeks post injury (wpi). In conclusion, our results have directly shown that RGC survival and axon regrowth are responsible for functional recovery after ONI in adult zebrafish.

## Introduction

Optic nerve injury often induces massive cell death and irreversible visual functional impairment in mammals, such as mouse 1], rat 2,3], rabbit 4], and cat 5]. Lower vertebrates, like quail 6], *Rana pipiens* 7] and *Litoria moorei* 8], however, can recover visual function due to retinal ganglion cell (RGC) survival. In goldfish, about 90% of RGCs survive and rapidly regrow axons to tectum about 2 weeks after axotomy 9]. Being a member of lower vertebrates and a model organism, zebrafish has excellent potential to regenerate RGC axon to tectum within 5 days after optic nerve crush (ONC) 10]. It can restore visual function at 20–25 dpi 11], comparing with 40 days for cichlid 12], 30–50 days for goldfish 13] and 16 weeks for sunfish 14]. However, whether RGC survival or neurogenesis is required for visual functional recovery is still a matter of controversy 15].

It is generally believed that multipotent retinal stem cells can produce new cells to replace dead ones after injury 16]. Results from light-lesion photoreceptor model 17,18], retina epimorphic and ablation model 19,20,21,22], and even whole retina destruction model 23,24] all indicated that Müller cells performed as multipotent retinal stem cells to form neuronal progenitors. Additionally, after a spinal lesion, olig2-positive (olig2^+^) progenitor cells in the ventricular zone proliferated slowly and generated motor neurons which integrated into the existing adult spinal circuitry for functional recovery 25]. Indeed, stem cells also exist in mammalian retina and some pioneers have tried to transplant stem cells into retina to protect neurons from reduction 26,27].

Besides, RGC survival and axon regrowth in adult zebrafish, facilitated by both intrinsic and extrinsic factors, have been observed in previous studies 10,15,28]. It seems that newborn RGCs are not necessary for regeneration as the fast regrowing axons of survived RGCs to target could get sufficient neurotrophic factors for soma survival. So it is interesting to see which prevails during regeneration. Is it RGC survival or RGC neurogenesis? Although previous studies stated that newborn RGCs are unnecessary for axon regeneration in other species, there was no convincing evidences showing changes in the number of RGCs 29,30]. As the current gold standard of RGC counting is retrograde labeling from tectum 31], we completely labeled RGCs from zebrafish tectum and observed whether newborn RGCs are important to visual functional recovery.

In general, we investigated three questions on visual functional recovery of adult zebrafish after optic nerve injury (ONI). First, do newborn RGCs appear and take part in regeneration? Next, does retina undergo inflammation if almost all RGCs survive after ONI? Finally, does myelin structure within retina keep integrity during visual functional restoration? Unraveling the mystery of visual functional recovery in adult zebrafish will shed new light on treatments for mammalian nerve injury.

## Methods

### Animal

Adult zebrafish of 5∼10 months with body lengths between 2.6∼3.2 cm were used. Fish with similar size were selected for each experiment before randomization. AB/WT, *Tg(coro1a:eGFP; lyz:DsRed)* [32], *Tg(olig2:eGFP)* [33] and *Tg(flk:GFP)* transgenic lines were used for different aims. Zebrafish were maintained at 28.5°C with a 14/10 h light-dark cycle and a 2 times/day diet. All animal manipulations were conducted in strict accordance with the guidelines and regulations set forth by the University of Science and Technology of China (USTC) Animal Resources Center and University Animal Care and Use Committee. The protocol was approved by the Committee on the Ethics of Animal Experiments of the USTC (Permit Number: USTCACUC1103013). All zebrafish surgery was performed under solution of tricaine methane-sulfonate (MS-222, Sigma) anesthesia, and all efforts were made to minimize suffering.

### Microsurgery

Optic nerve injury was operated similarly to others 34]. Briefly, after anesthesia in 0.03% solution of MS-222, zebrafish were put on a piece of wet tissue paper with left eye upward under a dissecting stereomicroscope (BeiTek, China). The connective tissue around eye was removed with jewelry #5 forceps (F.S.T, Switzerland) and the eye ball was pulled out of the orbit to expose the optic nerve and eye vessels. The eye artery and optic nerve were carefully separated with a round-tipped glass needle, then the optic nerve was crushed about 10 s with fine forceps (ONC model) or cut with Venus scissors (ONT model) (F.S.T, Switzerland). The right eye was kept intact to serve as an internal control in experiment group. Sham group was operated with all the procedures above except the final crush or cut step. The fish were returned to the system environment for various periods from 1 to 84 days.

### Retrograde labeling RGC

Fish were anesthetized in 0.03% MS-222, then fixed in a sponge gab and dripped MS222 solution through the mouth at a half concentration. The skin was slightly removed over the whole skull with sclerectome and dried with ear washing bulb. The skull was corroded with the acid-etching (Gluma Etch 35 Gel, Heraeus Kulzer, Germany) for 10 s, completely washed with saline and dried. In order to label the RGCs of left retina, a circle was first drawn around the right tectum with lightcuring bond (Durafill bond, Heraeus Kulzer, Germany) and the bond was fixed with 40 s blue light for catalysis (Power Blue Light Curing Unit, China). Thus skull outside the right tectum was protected from the following deeper corrosion. The right tectum was acid-etched again for 2 minutes to be completely corroded. The acid-etching solution was washed out with saline and dried. The malacic skull was carefully removed with forceps and the right tectum was exposed. Then a piece of gelfoam which had been soaked in DiI (N22880, invitrogen, USA) was put on the right tectum. After the dye covered the whole right tectum, the hole was closed with a sterile scale from the same animal. After that, lightcuring bond was put onto the scale and solidified with blue light for 40 s. At last, the surface bond was washed and the animal was waked up with system water. The fish were kept in 28.5°C environment for 5 days and fed 2 times/day. If the dye falls off within 5 days, the fish should be ruled out of the experiment.

For retrograde labeling at the optic nerve stump 35], a piece of Dextran crystal was put behind it. The space between eye and orbit was sealed with lightcuring bond to keep the dye staying longer.

### OMR behavior test

Concordance ratio after binocular ONC was collected at various time from 7 to 84 days, and all behavior experiments were conducted in the afternoon between 2 PM and 8 PM (Refer to 36] for detailed information about this method). Concordance ratio is the quotient of fish following grating time divided by the whole observation time (usually 1 minute).

### Injection BrdU, Isolectin IB4 and zymosan into eyeball

Injection of small volume (40–60 nl) does not induce ocular hypertension (Picospritzer III, Parker, USA). Ten mg/ml of BrdU solution (0.68% saline:DMSO = 1∶1) (Sigma Aldrich, USA) was injected into the eyeball once every 24 h. One mg/ml solution of Isolectin IB4 (I-21412, invitrogen, USA) was injected into eyeball. After one day of marking, retina whole-mount was imaged immediately after it separated from the eye. Twenty-five mg/ml of zymosan solution (diluted by 0.68% saline, Sigma Aldrich, USA) was injected into the eye vitreous cavity. On the one hand, to investigate if inflammatory cells could help RGC regeneration, zymosan was injected into vitreous cavity 3 days before ONT and retrograde labeling 37]. On the other hand, to study whether inflammatory cells exert negative effects on RGC survival, RGC was labeled with DiI as above and the dye was removed after 5 days absorption. The skull was sealed for another 5 days, then zymosan was injected to vitreous cavity.

### Retina whole-mount imaging

Zebrafish were kept in dark field for 2 hours before retina whole-mount imaging. The fish were anesthetized in ice water, an eye cup was made by removing the cornea with Venus scissors, and the space between retina and sclera was flushed with ice-cold 1× PBS to wash the pigment out. The dorsal position was marked and the optic nerve was cut at the disc. The retina was fixed in 2% paraformaldehyde (PFA, Sigma) for 10 min on ice and was washed 3×5 minutes with ice-cold PBS, then was flattened to a glass slide with retina ganglion cell layer (RGCL) upward and covered with 75% glycerin solution.

Whole retina was non-interlaced imaged under the 20× object lens (BX60, Olympus) with 1360×1024 pixels and each field had three different focuses. The depth of different focus was extended with IPP6 software (Media Cybemetics, USA) and a virtual retina was assembled with Photoshop CS2 (Adobe, USA). Three fields of 680×512 pixels (350 µm×264 µm = 0.092 mm^2^) were selected in each orientation of retina to analyze the average density of RGC, from which the total number of RGC can be calculated.

### Immunohistochemistry

After anesthesia in 0.03% MS-222, fish were immobilized in a gab of paraffin platform with ventral side up, perfused intracardially 38] with 0.68% saline for 1 min and then with 2% paraformaldehyde (pH 7.4, 1× PBS) for 2 min at a speed of 1 ml/min, followed by fixation in 2% paraformaldehyde (pH 7.4, 1× PBS) for 2 hours at room temperature (the retina was slightly fixed for 15 min specifically for Nav 1.6 immuniohistochemistry). Finally, the retina was washed three times in PBS (pH 7.4), equilibrated in 30% sucrose in PBS and 5 µm cryosections were cut (Leica, Germany).

Mouse anti-BrdU (Sigma) for detecting newborn cells was used at 1∶1000. Mouse anti-Nav 1.6 (Sigma) specifically labeling Ranvier node was used at 1∶1000. Custom-made rabbit anti-MBP (1∶1000, Abmart, China) antibody was prepared according to Woods (2006). All secondary antibodies were from Invitrogen, including goat anti-mouse alexa fluor 488, goat anti-mouse alexa fluor 568, donkey anti-mouse alexa fluor 647, donkey anti-rabbit alexa fluor 488 and goat anti-rabbit alexa fluor 635.

### Western blot

Each 4 retinae were lysed in 20 µl SDS sample buffer (ZFIN protocol), 10 µl of cell lysate was resolved by 12% SDS–polyacrylamide gel electrophoresis and the protein was transferred onto polyvinylidene difluoride membrane (Millipore, USA) for antibody recognition. Anti-MBP (as above) and anti-β-actin (Cell Signaling, USA) antibodies were used at 1∶1000. The secondary antibodies, horseradish peroxidase–conjugated goat anti-mouse or donkey anti rabbit were used at 1∶10000. The proteins were visualized with ECL detection kit (Sango, China) and imaged with LAS4000 biomolecular imager (GE, USA).

### Transmission electron microscopy

Animals were perfused as above and eyeballs were removed into primary fixative (2.5% glutaraldehyde in 0.1 M phosphate buffer, pH 7.4) overnight at 4°C. The next steps were followed by standard sample preparations for electron microscopy 39]. Image fields of myelin structure within retina were taken in the nerve fiber layer (NFL) by transmission electron microscope (JEM-1230, Japan).

### Statistical analysis

The data was analysed with one-way ANOVA (by Turkey's test) in GraphPad Prism version 4.0 (Prism, USA) and results were shown as mean ± SEM. The dots lying on the top of each column in histogram represent the sample size, *i.e.* n. The criterion of significance was set at P<0.05. *, ** and *** represent P<0.05, P<0.01 and P<0.001 respectively.

## Results

### 1 RGC survival after optic nerve injury

**Figure 1:**
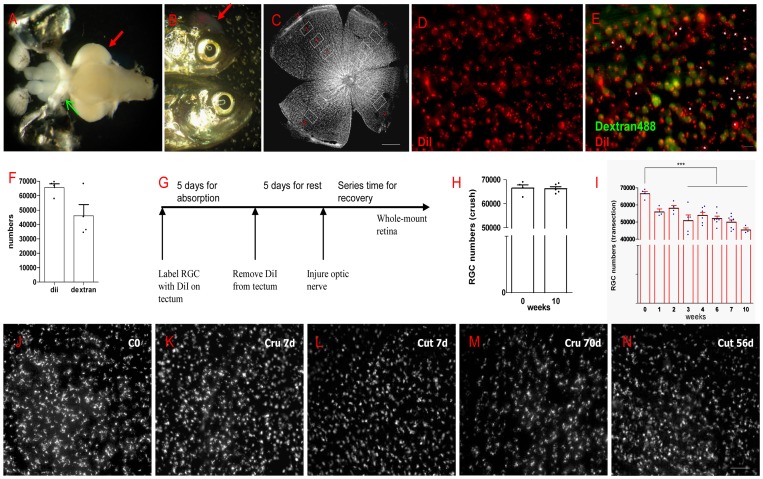
Most RGCs survived after ONI. (A) Two methods were used to label RGCs, the red arrow indicates DiI placed on tectum, the green arrow indicates Dextran 488 at optic nerve stump. (B) The red arrow shows lightcuring bond on the skull to prevent DiI lost. (C) The sampling of RGCs counting in the entire retina. (D) An example of DiI labeled RGCs. (E) Merge DiI and Dextran labeled RGCs in the same field. Asterisks indicate cells labeled by DiI but not by Dextran488. (F) Though the number of labeled cells has no significant difference (p>0.05) between DiI and Dextran, retrograde labeling with DiI is more reliable. (G) A scheme of DiI labeling in RGC survival experiment. (H) Almost all RGCs survived in ONC model after 10 weeks. (I) Significant RGCs lost began at 3 weeks (about 20% lost) but over 70% of RGCs were still alive at 10 weeks after ONT, p<0.001. (J-N) Representative pictures show RGCs at different time in ONC or ONT model. * indicates p<0.05, *** is p<0.001. Scale bar: 200 µm (C); 10 µm (E); 30 µm (J-N).

To observe the change of RGCs number, we first established a method to retrograde label RGCs completely from adult zebrafish tectum. As zebrafish optic nerves display absolute cross projection, it is necessary to check which method is better between retrograde labeling from the tectum or from optic nerve stump 35,40]. Figure 1A is the ventral view of the whole brain of adult zebrafish. Green arrow indicates retrograde labeling RGC at optic nerve stump with a piece of Dextran 488 crystal, and red arrow indicates retrograde labeling RGC from the tectum with DiI. As Figure 1B shows, red arrow in the upper fish indicates that some lightcuring bonds were sealing the hole of the skull to prevent DiI loss, with the lower one being control. By comparison, DiI needs 5 days to label all RGCs from tectum, and Dextran needs 1 day. In Figure 1D, fluorescent signals of DiI form a non-uniform pattern, while Dextran diffuses throughout cytoplasm and display pervasive fluorescence (green in Figure 1E). However, by analyzing RGC number (the counting method is shown in Figure 1C), we found that DiI labeled more RGCs than Dextran in the same fish operated by the same person (white asterisks in Figure 1E). Hence DiI is more reliable than Dextran labeling in zebrafish (Figure 1F). And Figure S1 shows nearly two thirds of DAPI positive cells in the NFL are also DiI labeled, which means DiI labeled almost all RGCs in retina.

In order to observe how many RGCs survived after ONI, retrograde labeling RGCs from tectum was performed first in normal fish, then optic nerve was injured and the number of RGCs within whole retina was recorded at different time points (Figure 1G). To prevent regenerating axons from touching dye and confusing the result, DiI gelfoam should be removed after being absorbed completely for 5 days and the fish were allowed to recover 5 days before ONI (Figure 1G). In ONC model, ONI barely induced loss of RGCs. Even after 10 weeks, RGC number showed no decrease in comparison with normal fish (66507±1358 in normal group vs. 66201±858 in 10 wpi, 99.5% survived, Figure 1H). Wondering the reactions in ONT, we injured optic nerve severely and made long-term observations. In the first 2 weeks, RGC number decreased about 15% (55884±1785 in 1 wpi and 58040±1654 in 2 wpi), with about a 25% decrease at 3 wpi (50785±3379, p<0.001) and with 75% remaining until 7 wpi (49908±1363, p<0.001, Figure 1I). At 10 wpi, the number of RGCs still remained at about 70% (45346±1277, p<0.001). The examples of RGC density at different time points after ONI are shown in Figure 1J-N. All these results indicate a strong ability of zebrafish RGCs to survive after ONI.

### 2 RGC regrowth after optic nerve injury

**Figure 2:**
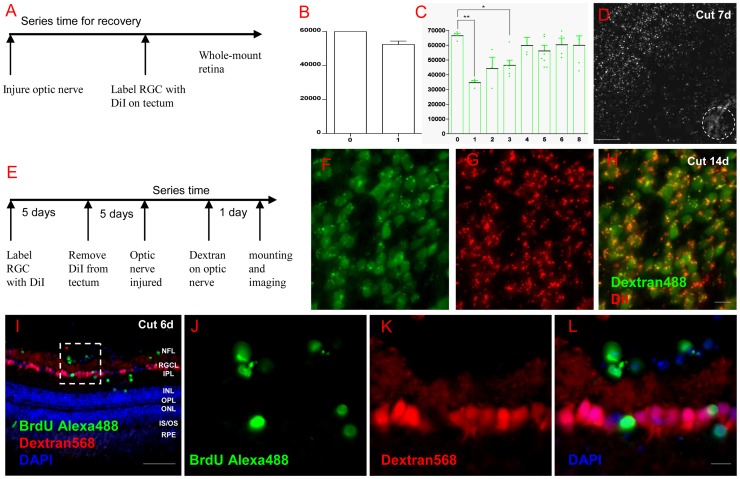
RGCs regenerated quickly and no new RGCs were found. (A) Scheme of DiI labeling in RGC regeneration experiment. (B) In ONC model, over 90% of RGCs regenerated to their targets in the first week. (C) In ONT model, over 50% of RGCs regrew axons to tectum at 1 week post lesion (p<0.01, compared to normal) and the number was still significant compared to the normal animals after 3 weeks (n = 6, p<0.05). After 4 weeks, RGCs number was about 90% of normal fish and was no significant difference compared with normal (n = 3, p>0.05). (D) Periphery RGCs regrew axons to tectum more quickly than central ones during at the first week. (E) A scheme of DiI labeling RGC survival and Dextran labeling RGC regeneration in the same retina. (F-H) After 2 weeks post ONT, Dextran488 only marked RGCs were rarely found in the whole retina. (I) 6 days after ONT, BrdU^+^ cells could be found in the GCL and INL, but those cells did not co-localize with Dextran568 labeled RGCs. (J-L) Details of the white square in (I). Abbreviations: Neurofiber layer (NFL), retinal ganglion cell layer (RGCL), inner plexiform layer (IPL), inner nuclear layer (INL), outer plexiform layer (OPL), outer nuclear layer (ONL), photoreceptor inner segment/outer segment (IS/OS), and retinal pigment epithelium (RPE). * indicates p<0.05, ** is p<0.01. Scale bar: 100 µm (D); 50 µm (I); 10 µm (F-H, J-L).

Next, we modified the time of DiI labeling as shown in Figure 2A to observe how many RGCs regenerated to the target after ONI. Surprisingly, over 90% of RGCs arrived at tectum in the first week after ONC (Figure 2B). Considering that some RGCs arrived at the tectum but did not absorb enough dye at that time, it is possible that all of the surviving RGCs have regenerated to the tectum at the first week in ONC model. We then performed severely injured ONT experiment. As Figure 2C shows, over 50% of RGCs regrew to the tectum in the first week (34745±1344 regenerated in 1 wpi, p<0.01) and about 70% of RGCs by 3wpi (46448±3373, p<0.05). RGC number reached a platform after 4 weeks without significant difference from control (59863±5415, nearly 90% of normal group, P>0.05). Interestingly, the surviving RGCs in retina regenerated in a concentric manner with peripheral RGCs regenerating first and central RGCs later in the first week (Figure 2D). This is consistent with the classic phenomenon that axon regenerates easily when the injury is far away from the soma 2,31].

**Figure 3:**
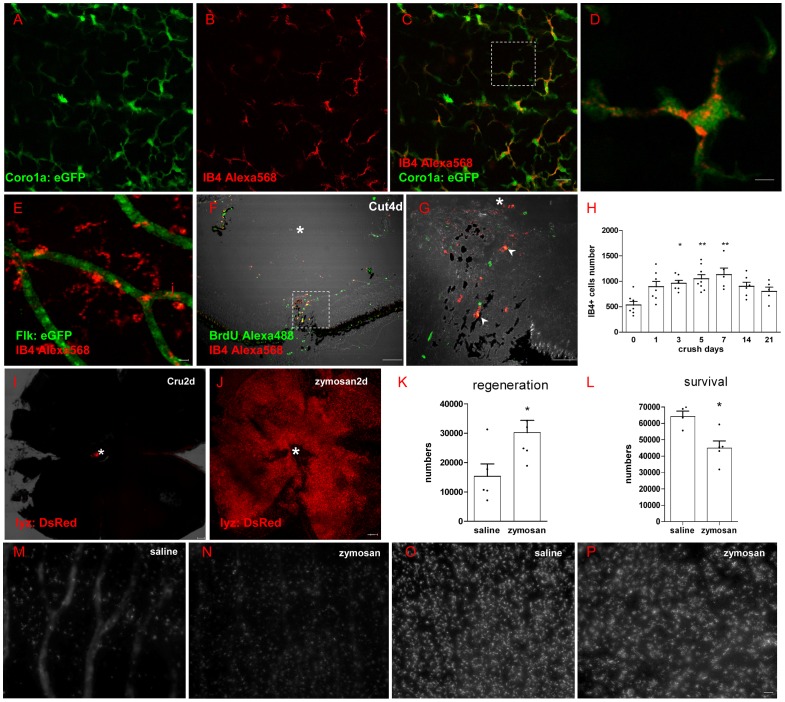
Inflammation has dual-roles during regeneration. (A-D) IB4^+^ cells in retina are colocalized with coro1a:eGFP in *Tg(coro1a:eGFP; lyz:DsRed)* fish, which means IB4^+^ cells mark microglia cells in zebrafish retina. (D) Shows the detailed view of square in (C). (E) IB4^+^ cells could not colocalize with flk:GFP in transgenic *Tg(flk:GFP)* line. (F-G) BrdU marker shows that some IB4^+^ cells are newborn in retina (arrowhead in G), * indicates the lens. (H) IB4^+^ cells number in all layers of retina changed with time, increasing significantly 3 days to 7 days after ONC, then decreasing to normal level after 14 days. (I-J) Zymosan induces acute neutrophil/macrophage infiltration into retina, * indicates optic disc. (K, M and N) Neutrophil/macrophage infiltration into retina could increase the number of regenerated RGCs in the first week after ONT, but decrease RGCs survival after 2 weeks (L, O and P). * indicates p<0.05, ** is p<0.01. Scale bar: 10 µm (A-C, M-P); 2 µm (D); 30 µm (G), 100 µm (F, I and J).

At 2 weeks after ONT, we double retrograde labeled the retina, with DiI labeling surviving RGCs first and Dextran488 labeling regenerated RGCs later (scheme see in Figure 2E). Cells, single labeled by Dextran, were seldom found (Figure 2F-H, n = 5). Though some BrdU^+^ cells in the RGCL may indicate that newly generated cells exist in this area, they are not superimposed on RGCs labeled by Dextran568 (Figure 2I-2L, also could see Figure 3G). So RGCs regenerate to tectum quickly without the participation of newborn RGCs.

### 3 Temporary activation of inflammatory cells

As microglia showed long-lasting increase and the majority of RGCs died after ONI in rat retina, we considered that perhaps the inflammatory responses in zebrafish might also affect RGCs survived after ONI 41]. We first labeled microglia in retina with isolectin IB4, which was used to mark microglia in mouse 42] and quail 43]. It has been shown that microglia in CNS of *Tg(coro1a:eGFP; lyz:DsRed)* line can be labeled with eGFP but not DsRed 32]. Figure 3A shows microglia (eGFP) in normal fish retina, while Figure 3B shows IB4-labeled microglia at the same locus, and the merged image in Figure 3C shows the co-existence of IB4^+^ and eGFP^+^ cells. Figure 3D is a whole view picture showing IB4^+^ signals on the membrane of microglia. However, IB4 does not label blood vessels (Figure 3E) in zebrafish as it does in mouse 42].

Next, we used IB4^+^ to count changes in the number of microglia in retina after ONI. The number of IB4^+^ cells in all layers of retina was increased significantly on the third day after ONC (p<0.05), returning to normal level 14 days later (Figure 3H). Some IB4^+^ cells are labeled by BrdU 44], indicating that microglial proliferation might contribute to the increased number of IB4^+^ cells (Figure 3F-G). Additionally, this result also explains why BrdU^+^ cells in the RGCL are not RGCs (Figure 2I-2L).

Inflammation in retina has been shown to help RGC regeneration in mammals 37]. Wondering if it is the same with zebrafish, in preliminary experiments we induced inflammation in zebrafish retina by injecting zymosan into the space of vitreous cavity. Figure 3I shows that only a few neutrophils and macrophages emerged in the optic disc at two days after ONC (also see Figure S3). Two days after zymosan injection, however, large amounts of neutrophil/macrophage were recruited to the whole retina 45] (Figure 3I-J). In regeneration experiment, zymosan was injected 3 days before ONT and the number of regenerated RGCs was much larger than the control group at 1 wpi (Figure 3K, M and N, 30236±4228 in zymosan group vs. 15322±4314 in saline group, p<0.05). However, in survival experiment operated on fish with intact optic nerve, the number of RGCs in the zymosan injection group was significantly smaller than the control group after 14 days (Figure 3L, O and P, 44904±4368 in zymosan group vs. 64253±3266 in saline group, p<0.05). Hence, inflammation in zebrafish retina is beneficial to RGC regeneration in the early period while harmful to RGC survival in the later stage.

### 4 Oligodendrocytes survival and myelin structure integrality in retina after ONI

As zebrafish has myelin in the retina while mammals do not, we are curious to know whether ONI impairs retinal myelin in zebrafish. Though it has been indicated that olig2^+^ cells in the NFL are oligodendrocytes in adult zebrafish retina 46], the eGFP only appears in the soma of oligodendrocytes 23,33]. Thus, we still wonder whether the olig2:eGFP cells are mature oligodendrocytes in the NFL of adult zebrafish retina.

**Figure 4:**
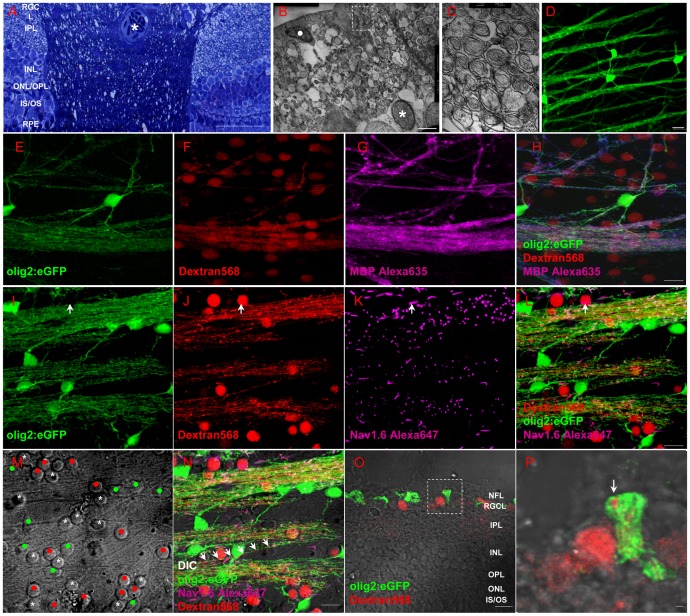
Oligodendrocytes exist within retina of adult zebrafish. (A) Toluidin blue staining shows myelin structure through the lamina cribrosa into the retina. (B-C) TEM images show that the axons of RGCs are specially and loosely wrapped by myelin. (D) Olig2:eGFP cells in retina have perfect structure of oligodendrocyte cells that project several processes to vicinal axons in *Tg(olig2:eGFP)* transgenic line. (E-H) Combination of Dextran568 labeled RGCs and MBP staining show olig2^+^ eGFP expression on mature oligodendrocytes in NFL. Notice that only the myelin ensheathed axons are MBP positive. (I-N) With Ranvier node on myelin, oligodendrocytes in zebrafish retina are in mature state. Arrow in (I-L) points out an axon hillock near RGC soma, and it should be noticed that olig2^+^ process dose not wrap this area. Red dots in (M) indicate RGCs, green dots are olig2^+^ cells and * indicates non-labeled cells, a row of arrows in (N) show an axon is wrapped by olig2^+^ process. (O) Crossing section indicates that axon retrogradely labeled by Dextran568 is wrapped by olig2^+^ eGFP. (P) Details of the white square in (O). Scale bar: 50 µm (A); 2 µm (B); 200 nm (C); 10 µm (D-O); 1 µm (P).

First, myelin structure was found in the disc area by toluidin blue staining (Figure 4A). Its ultrastructure was found in NFL (Figure 4B) and details were shown in Figure 4C. Different from optic nerve or retina myelin in other vertebrates 47], this myelin is single layered around RGC axon without nuclei nearby (Figure 4C), meaning that it is wrapped by oligodendrocytes. Olig2^+^ cells in the NFL of whole-mounting retina in *Tg(olig2:eGFP)* fish line has perfect process to wrap axon and the myelin structure in Figure 3C is labeled by GFP (Figure 4D). Further evidence from IHC is shown in Figure 4E-N. Green in Figure 4E is GFP fluorescence in *Tg(olig2:eGFP)* fish line, red in Figure 4F is RGC body and axon labeled by Dextran 568 from optic nerve stump, violet in Figure 4G is MBP staining, and the merged picture is shown in Figure 4H. It is clear that olig2^+^ area completely overlaps with MBP^+^ area, meaning that olig2^+^ cells protrude branches to wrap axons 48]. When we combine olig2^+^ fluorescence (Figure 4I) with RGC axon (Figure 4J) and Ranvier node (Figure 4K), there is no doubt that olig2^+^ process ensheathes RGC axons in a mature state (Figure 4L), except the axon hillock (arrow in Figure 4I-L, and large images are shown in Figure S4). An example of a single axon wrapped by olig2^+^ process is show in Figure 4N by a row of arrows. For further confirmation, RGCs were retrograde labeled with Dextran568 in olig2:eGFP line and retina was cross sectioned for observation (Figure 4O). The arrow in Figure 4P definitely shows that RGC axon is wrapped by olig2^+^ process.

**Figure 5:**
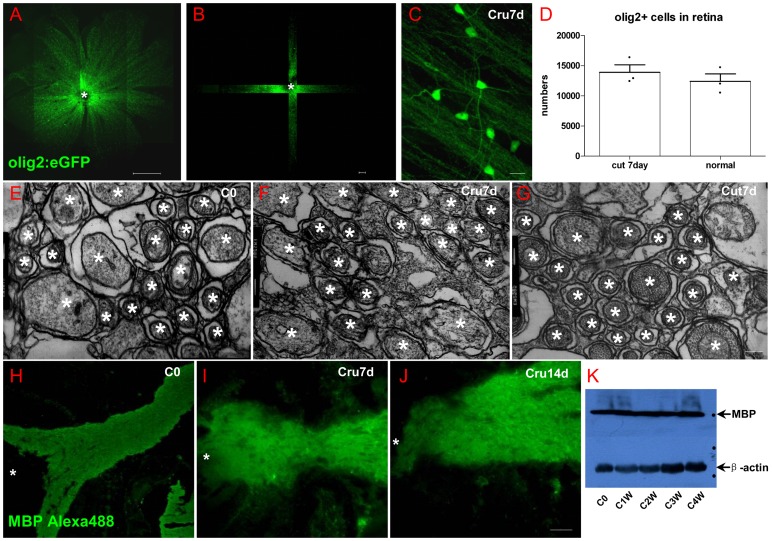
Oligodendrocytes in retina did not decrease after optic nerve injury. (A) Scan the whole retina with 10× objective of LSM710. (B) Cruciated scan in 3D way for counting the destiny of olig2+ cells. (C) The processes of oligodendrocytes keep integrality after ONI. (D) Oligodendrocytes number did not decrease at one week after ONT. (E-G) TEM images also show that myelin structure within NFL keeps integrity in ONC or ONT model within the first week. (H-J) Both IHC and western blot results (K) indicated that MBP in retina was not decreased after optic nerve injury, * indicates lens; Scale bar: 200 µm (A); 50 µm (B, H-J), 10 µm (C); 200 nm (E-G).

We then considered whether myelin structure or oligodendrocytes are influenced by ONI in adult zebrafish. Since only a half of regenerated RGCs regrow axons to the tectum in the first week, we decided to observe myelin structure and oligodendrocyte at the first week after ONT. As the NFL in disc is thicker than peripheral retina, we scanned the whole retina with a 10× objective lens to get the area of retina (Figure 5A) and then cruciated scan it in 3D ways (63× objective lens, 50 slices, interval  = 1 µm, LSM710, Zeiss) to get the density of olig2^+^ cells (Figure 5B). Not only the process of olig2^+^ cell stays intact (Figure 5C), but also the total number of olig2^+^ cells in NFL is not influenced by ONT (12407±1229 in normal group and 13897±1243 in injured group, Figure 5D). The ultrastructure of myelin in NFL of normal fish (Figure 5E) and injured fish (crush 7 days in Figure 5F and cut 7 days in Figure 5G, n = 3) is also not significantly impaired. Asterisks show that RGC axons are completely wrapped by myelin (Figure 5E-G). MBP staining (Figure 5H-J) and western blotting result (Figure 5K) also show no MBP decrease after ONI. The collective data in Figure 4 and Figure 5 demonstrate that oligodendrocyte number and myelin structure in NFL of adult zebrafish have no detectable change under the condition of ONI.

### 5 Oligodendrocytes survival are beneficial to visual functional restoration

**Figure 6:**
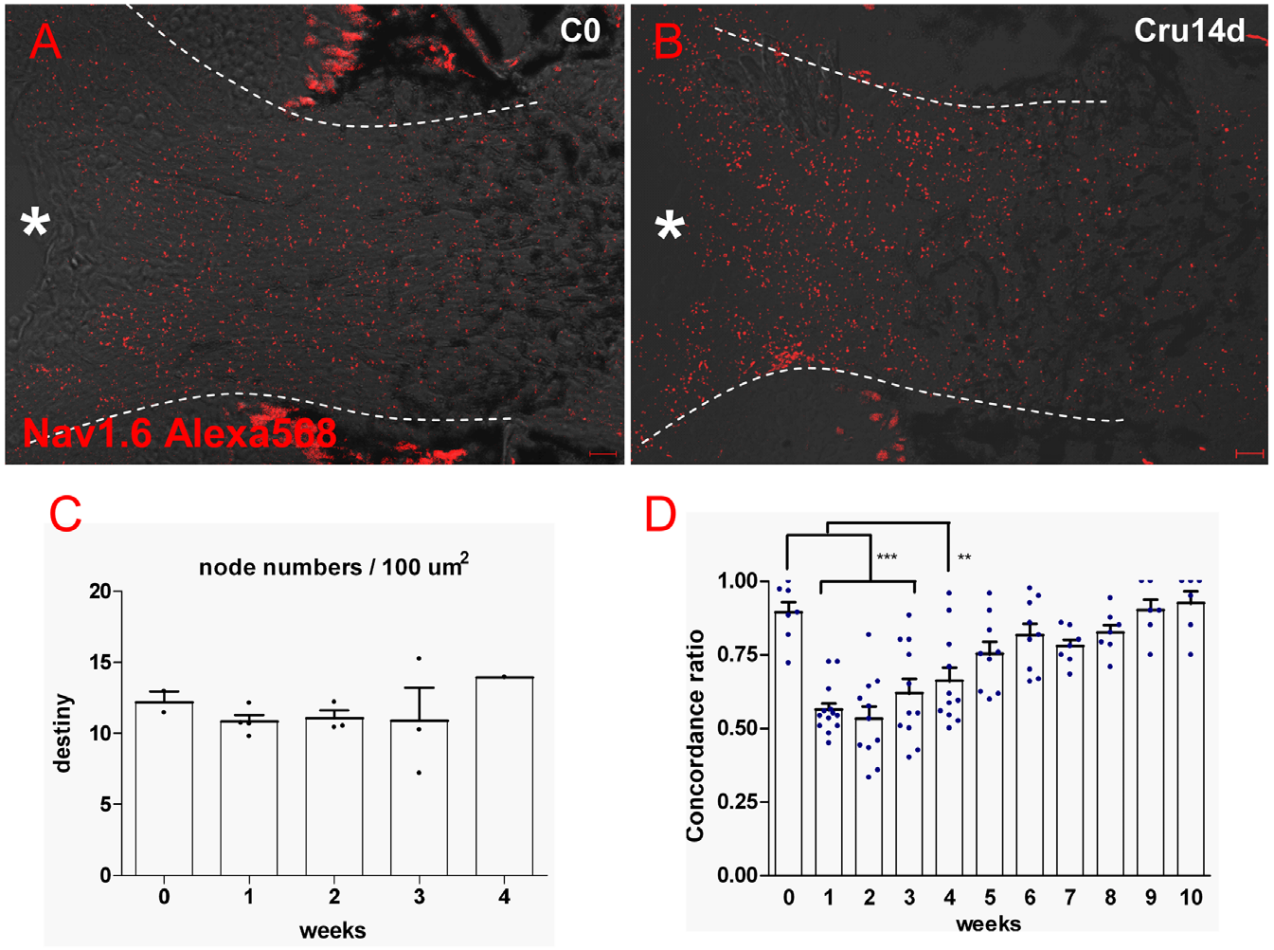
(A, B) The density of Ranvier node in optic disc is not influenced by optic nerve injury. (C) Quantitative result shows Ranvier node density in disc is not decreased following ONI. (D) OMR result shows gradual recovery of visual function. After 5 weeks of binocular optic nerve crush, concordant ratio returns to normal level (p>0.05) and visual function is completely recovered at 9 wpi (100% recovery). ** indicates p<0.01, *** is p<0.001. Scale bar: 10 µm (B).

As most RGCs survive and oligodendrocytes keep structural integrality, we considered whether Ranvier node also keeps its integrality as action potential conduction is the basis of visual function. For analysis of Ranvier node in retina, we chose the optic disc where all RGC axons must pass by for projection to the brain. Neither qualitative nor quantitative analysis of Ranvier node density shows significant difference between crushed and control fish (Figure 6A-C).

We further conducted OMR behavioral experiment to evaluate the visual function after ONC. To avoid the influence from binocular vision 49], binocular crush was conducted in this experiment. In Figure 6D, concordant ratio in normal fish is near 1, then decreases to 0.5 in the first 2 weeks after binocular crush (p<0.001), meaning fish swim randomly. At 5 weeks after injury, the concordant ratio is not different from normal group (p>0.05) and all visual functions recovered completely at 9 wpi. This means that visual functional recovery benefits from RGC survival, axon regeneration and node structure integrity.

## Discussion

Trying to solve the controversy of whether RGCs neurogenesis is required for visual functional recovery in ONI model, we labeled RGCs from the tectum of adult zebrafish to monitor the number of surviving RGCs and RGC regeneration. Results showed that instead of proliferation of RGCs, survival and regeneration of original RGCs are responsible for visual functional recovery. As few RGCs died during regeneration, microglia/macrophage was activated temporarily. Meanwhile, we certified that olig2^+^ cells in RGCL of adult zebrafish are mature oligodendrocytes and have no detectable change after ONI.

Retrograde labeling from tectum is very important in the study of RGC survival. Dextran labeling from the optic nerve adds an extra injury to RGCs, so it is not applied to study the survival of RGC after ONC 35]. HE staining 50] and antibody staining 24] are not a widely-accepted procedure for counting RGCs numbers, and they could not distinguish the original RGCs from regenerated ones. Transgenic line with all RGCs marked has not been constructed 51,52]. Referring to goldfish 53], we retrograde labeled RGCs from the tectum in adult zebrafish. With the help of acid-etching, it is very convenient to open the skull and minimize the harm to the brain. With light-curing bond to seal the hole, both the rate of animal survival and dye retention after retrograde labeling could reach almost 100%. We have identified that DiI on the tectum over 5 days can label all RGCs which have arrived tectum 54]. The number of Dextran labeled RGCs was less than DiI labeled RGCs (Figure 1F), in that Dextran was diluted once zebrafish were placed in the water.

It is not surprising that most RGCs survive as many studies have shown that survival molecules are increased during regeneration 15] and *in vitro* culture experiments show little RGCs death after ONT 55]. What is interesting is that over 50% of surviving RGCs regrew their axons to the target at the first week after ONT. Considering the distance from transection gap (middle in optic nerve) to tectum, which is more than 1 mm 38], the mean speed of axon regrowth during regeneration is about 200 µm/day. Figures S2A-B show that few RGCs regrew to the tectum within 5 days, meaning a higher axon regrowing speed than fin regeneration (200 µm/day) 56]. It is plausible that the quick regrowth of axon to tectum protects RGC soma from apoptosis by re-gaining trophic support from the target.

Newborn RGCs were seldom found in the case of ONI, let alone in the ONC model. However, it seems that there are slightly more regenerated RGCs than surviving ones in the ONT model at the late stage (after 6 wpi) of regeneration (Figure 1I vs. Figure 2C). Though new RGCs were rarely found in the first 2 weeks after ONT (Figure 2F-L), we could not exclude the possibility that quite a few new RGCs form during such long period of regeneration [57]. We analyzed the regenerated and surviving RGCs at the same time. Taking 4 and 6 wpi for example, these two sets of data have no significant differences (p value is 0.2234 and 0.0526 respectively). Considering that visual function was restored by 5wpi, it is reasonable to believe that RGC proliferation is not essential for such functional recovery in ONT model. Though RGCL can regenerate in a whole retina injury model induced by ouabain 23], only 75% of somas (ganglion and amacrine cells) are recovered in RGCL at 60 dpi and visual function is recovered at 98 dpi. Besides, the differentiation of Müller cells is quantity-and time-dependently decided 22]. Only a large amount and acute death of photoreceptor cells could initiate Müller glia proliferation. The neurogenesis trigger signal in different injury models still remain to be elucidated.

The striking ability of axon regeneration in teleost has been well demonstrated from intrinsic factors and extrinsic factors 15,58]. Our results in Figure 2D showed that RGCs regrowing to the target area faster were mainly located in the peripheral zone of the retina, which implies the intrinsic factors are very important for axon regeneration. While RGC death could induce a long prolonged inflammation in avian 43] and mammalian 59] retina, this was not the case in zebrafish. Though few RGCs died in zebrafish, the transitory inflammation may play dual-roles to enhance RGC regeneration at early stage and fade away later to alleviate the harmful effects to normal RGC survival 37].

Visual functional recovery in adult zebrafish includes RGC survival, axon regrowth and oligodendrocyte remyelination. Myelin-wrapped RGC axons within retina are found in some vertebrates but not in mammals 60,61,62]. After perfused intracardially, the process of oligodendrocytes expressed olig2:eGFP extensively. Combining TEM and IHC results, we certify that mature oligodendrocytes exist in the retina of adult zebrafish (Figure 4) and ensheath RGCs axon in a single lamina way (Figure 5). The existence of Ranvier nodes indicates myelin within retina could help action conduction. However, where are these oligodendrocytes from and why are they present? Why are RGC axons only wrapped in a single myelin layer? What mechanisms regulate such loose wrapping? These important questions remained to be solved.

Finally, complete visual functional recovery in our study is slower than that from previous reports 11]. As both left and right optic nerves were injured in our behavioral experiment, it may take more time to restore visual function. Nevertheless considering that visual functional recovery takes 4 months in goldfish 13], recovery in zebrafish is still fast.

In conclusion, our results demonstrate that adult zebrafish adopt RGC survival and axon regeneration as the major strategy for visual functional recovery after ONI. RGCs proliferation is not required, which is different from spinal cord restoration 25] and photoreceptor recovery 16].

## Supporting Information

Figure S1 **Number of DiI labeled RGCs (A) is about two third of DAPI numbers (B) in whole retina.** Scale bar: 40 µm (B).
(TIF)

Figure S2 **(A) Regenerated RGCs could not be found at 5 days after ONT, the large view is shown in (B).** Scale bar: 200 µm (A); 50 µm (B).
(TIF)

Figure S3 **Neutrophils (lyz: DsRed) were rarely found in the retina except in the disc during the first 3 days after ONC (A) and then disappeared at 7 dpi again (B).** Scale bar: 30 µm (B).
(TIF)

Figure S4 **Myelin process does not wrap axon at the site of axon hillock (arrow).** These arrows are the same in Figure 4I-L. Scale bar: 2 µm (D).
(TIF)
