# Supplementary figures and images for: Neurogenesis of Retinal Ganglion Cells Is Not Essential to Visual Functional Recovery after Optic Nerve Injury in Adult Zebrafish
Source: PLoS One. 2013 Feb 20;8(2):e57280. doi: 10.1371/journal.pone.0057280 (PMC3577741; doi:10.1371/journal.pone.0057280)

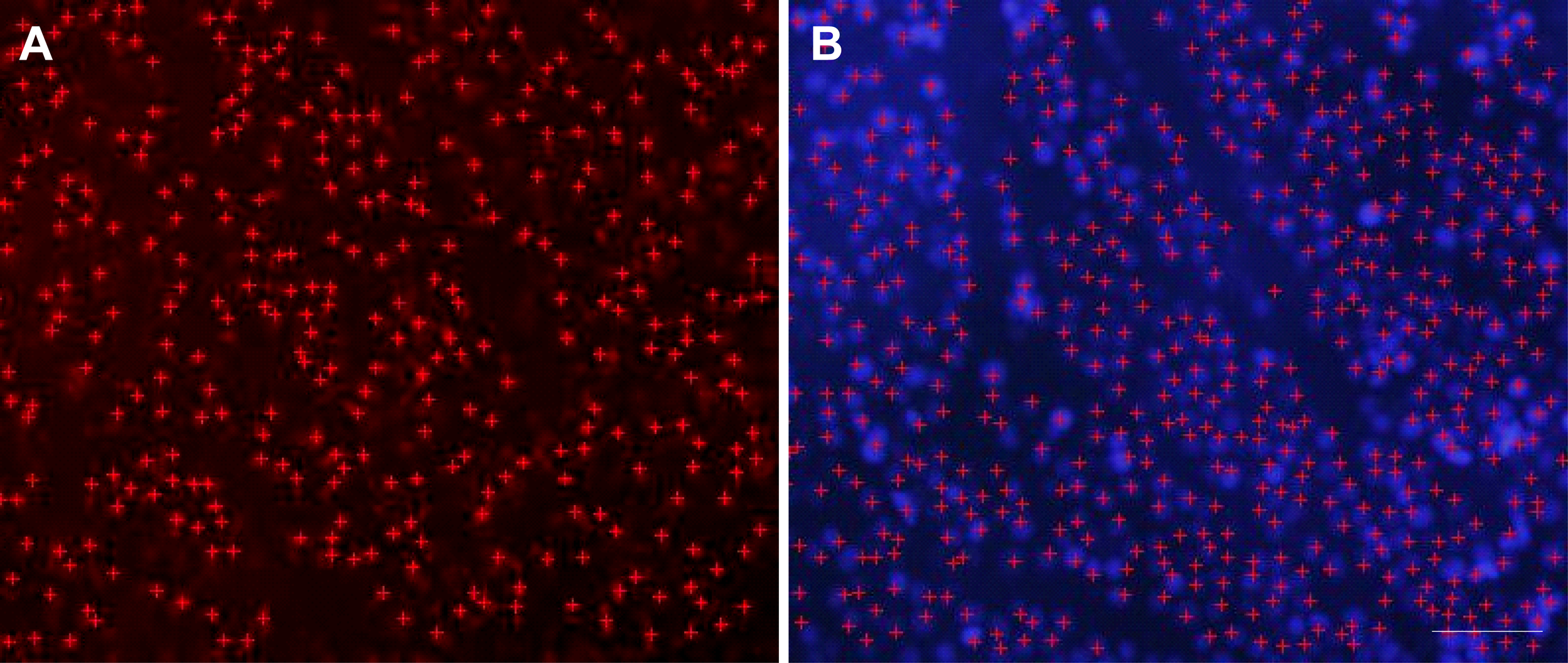

Supplement: Figure S1 — Number of DiI labeled RGCs (A) is about two third of DAPI numbers (B) in whole retina. Scale bar: 40 µm (B). (TIF) [file pone.0057280.s001.tif]

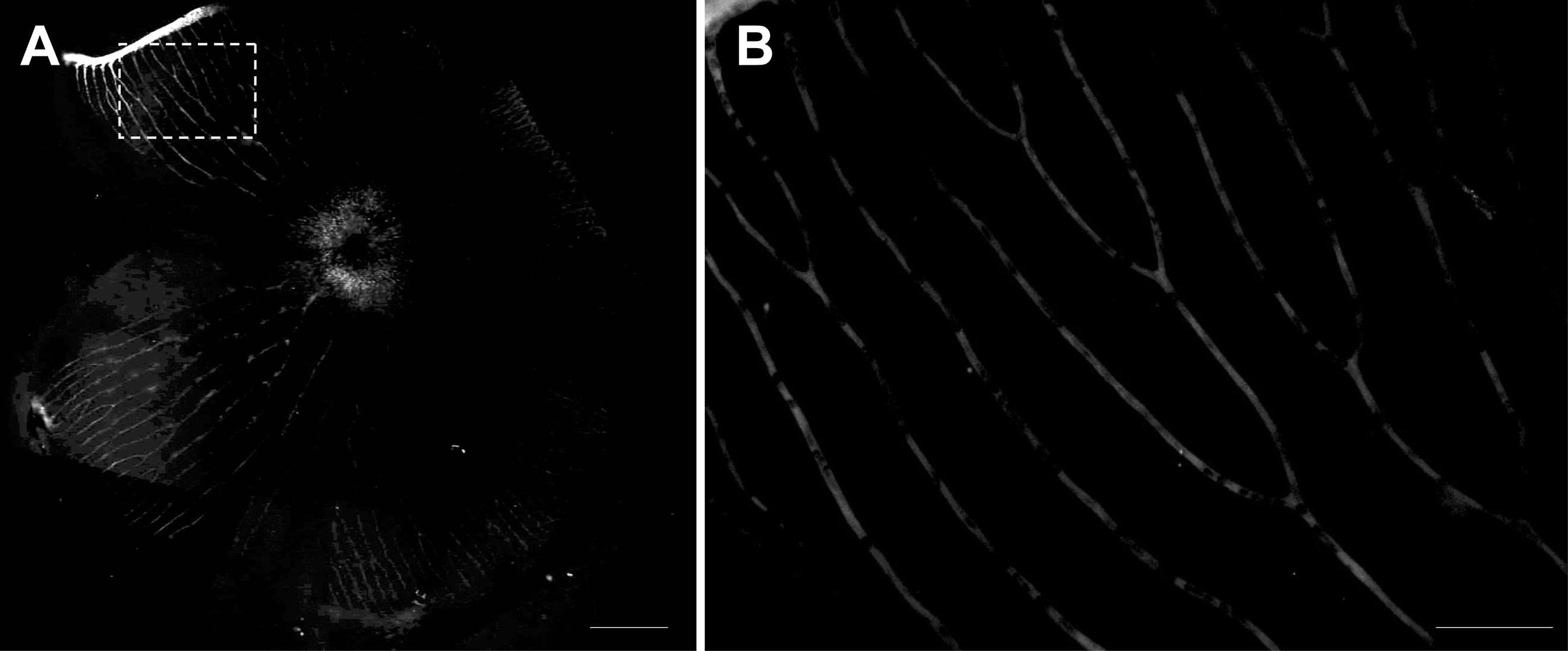

Supplement: Figure S2 — (A) Regenerated RGCs could not be found at 5 days after ONT, the large view is shown in (B). Scale bar: 200 µm (A); 50 µm (B). (TIF) [file pone.0057280.s002.tif]

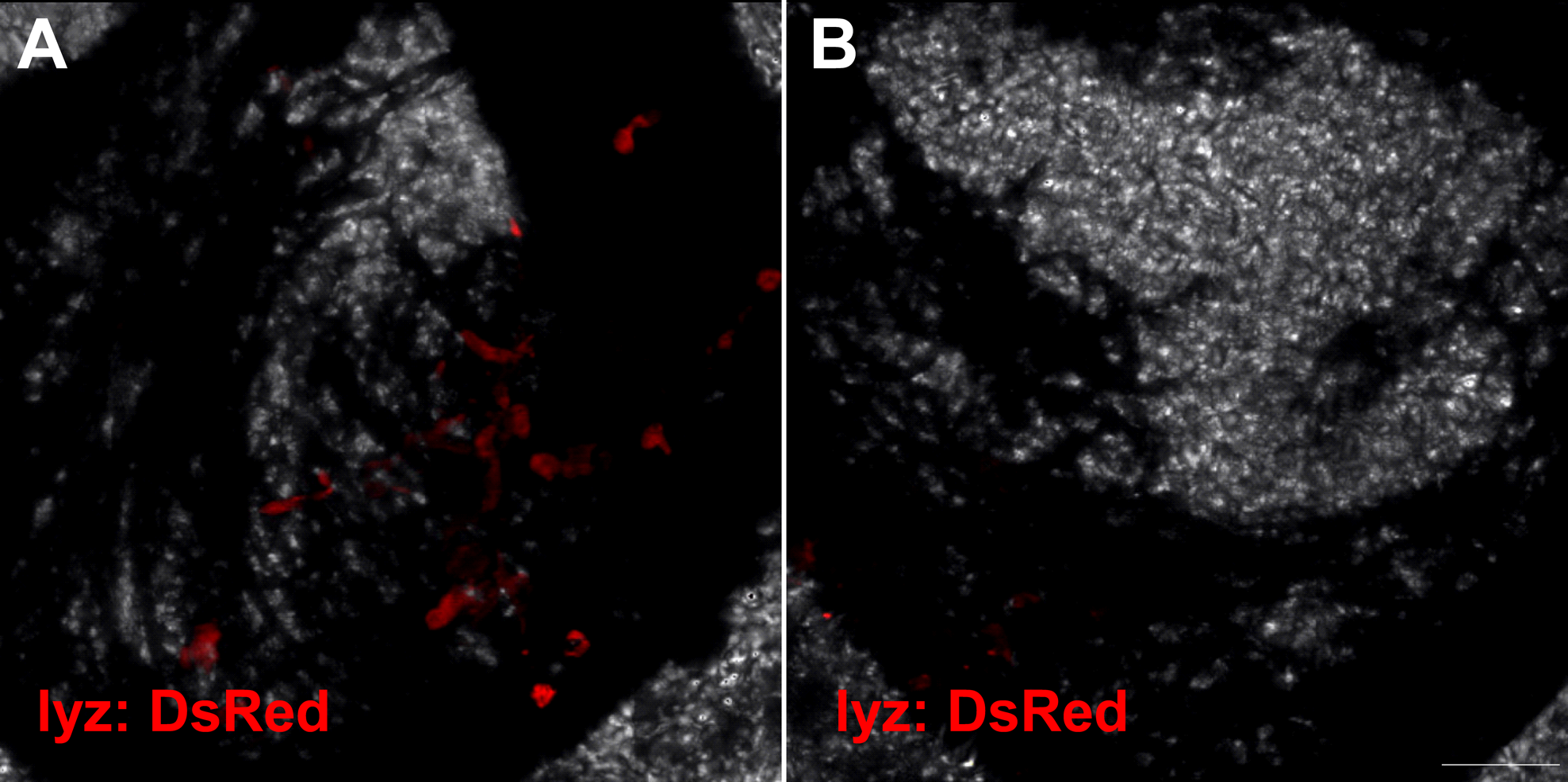

Supplement: Figure S3 — Neutrophils (lyz: DsRed) were rarely found in the retina except in the disc during the first 3 days after ONC (A) and then disappeared at 7 dpi again (B). Scale bar: 30 µm (B). (TIF) [file pone.0057280.s003.tif]

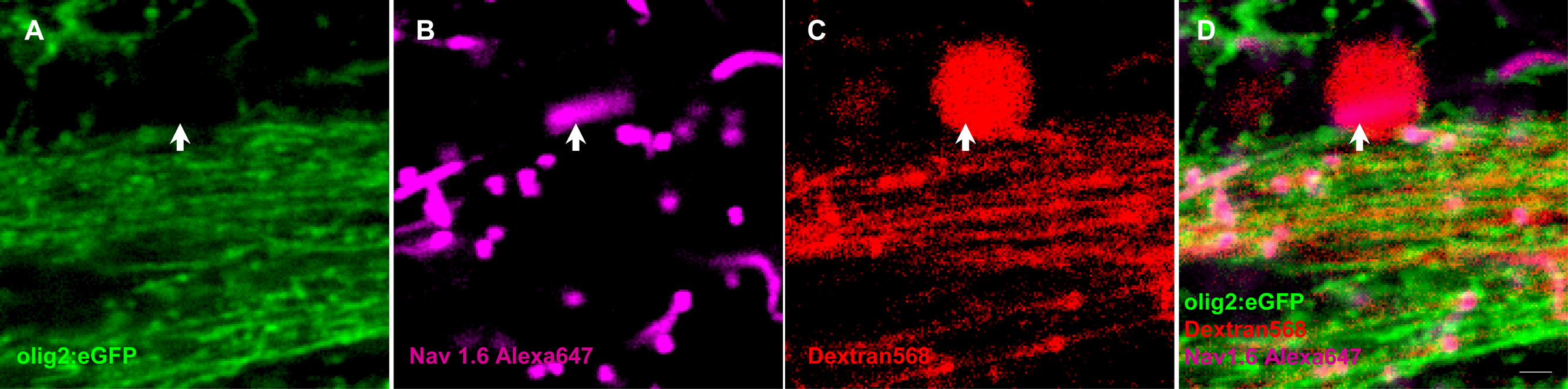

Supplement: Figure S4 — Myelin process does not wrap axon at the site of axon hillock (arrow). These arrows are the same in Figure 4I-L. Scale bar: 2 µm (D). (TIF) [file pone.0057280.s004.tif]
